# Investigating the Role of Dahuang in Hepatoma Treatment Using Network Pharmacology, Molecular Docking, and Survival Analysis

**DOI:** 10.1155/2022/5975223

**Published:** 2022-07-15

**Authors:** Bin Yu, Maoru Wang, Hui Xu, Rongrong Gao, Yuanying Zhu, Hong Ning, Xiaoyu Dai

**Affiliations:** ^1^Department of Pharmacy, Mianyang Central Hospital, School of Medicine, University of Electronic Science and Technology of China, Mianyang 621000, China; ^2^School of Pharmacy, Collaborative Innovation Center of Advanced Drug Delivery System and Biotech Drugs in Universities of Shandong, Key Laboratory of Molecular Pharmacology and Drug Evaluation, Yantai University, Yantai 264005, China; ^3^Department of Drug Dispensing, The Third Hospital of Mianyang, Mianyang 621000, China; ^4^Department of Nephrology, Mianyang Central Hospital, School of Medicine, University of Electronic Science and Technology of China, Mianyang 621000, China

## Abstract

Hepatoma is one of the most common malignant tumors. The incidence rate is high in developing countries, and China has the most significant number of cases. Dahuang is a classic traditional antitumor drug commonly used in China and has also been applied to treat hepatoma. However, the potential mechanism of Dahuang in treating hepatoma is not clear. Therefore, this study is aimed at elucidating the possible molecular mechanism and key targets of Dahuang using methods of network pharmacology, molecular docking, and survival analysis. Firstly, the active ingredients and key targets of Dahuang were analyzed through public databases, and then the drug-ingredient-target-disease network diagram of Dahuang against hepatoma was constructed. Five main active components and five core targets were determined according to the enrichment degree. Enrichment analysis demonstrated that Dahuang treated hepatoma through the multiple pathways in cancer. Additionally, molecular docking predicted that aloe-emodin and PIK3CG depicted the best binding energy. Survival analysis indicated that a high/ESR1 gene expression had a relatively good prognosis for patients with hepatoma (*p* < 0.05). In conclusion, the current study results demonstrated that Dahuang could treat hepatoma through a variety of active ingredients, targets, and multiantitumor pathways. Moreover, it effectively improved the prognosis of hepatoma patients. ESR1 is the potential key gene that is beneficial for the survival of hepatoma patients. Also, aloe-emodin and beta-sitosterol are the two main active crucial ingredients for hepatoma treatment. The study also provided some functional bases and references for the development of new drugs, target mining, and experimental animal research of hepatoma in the future.

## 1. Introduction

Hepatoma has become one of the major diseases threatening human health and hindering social development [1, 2]. According to the “Global Cancer Report 2020,” the global incidence of hepatoma has reached 910,000, and the newly increased incidence of hepatoma in China is 410,000. Therefore, the prevention and treatment of hepatoma have become a research hotspot. Currently, effective treatment schemes for hepatoma mainly include surgical resection, liver transplantation, radiotherapy, chemotherapy, and targeted therapy [3, 4]. Advanced hepatoma patients are primarily treated with chemotherapy and targeted therapy. Although such chemical drugs improve the cure rate of cancer, they exhibit significant toxic side effects and drug resistance [5, 6]. Therefore, it is necessary to explore and develop new drugs for hepatoma treatment. According to the clinical manifestations of hepatoma, traditional Chinese medicine (TCM) considers it to be a disease of “Accumulation” and “Swelling.” Moreover, TCM has been widely used in treating hepatoma in clinical practice for several years [7–9].

Dahuang, also known as rhubarb, is the rhizome of the *Rheum palmatum* plant, Tanggu giant Dahuang, or medicinal Dahuang [10]. Dahuang is widely used as a medicinal herb in China and other Asian countries for thousand years [11]. However, the Dahuang, in Europe and the Middle East, often refers to other varieties of Dahuang that have red stems and fragrant air and are used in food [12]. Modern pharmacological studies have shown that Dahuang has specific effects on pancreatic fibrosis, liver protection, and cardiovascular diseases and may display anti-inflammatory, antiviral, and antitumor action [13–19]. In recent years, increasing reports have described the antihepatoma effects of Dahuang. However, as traditional Chinese medicine, Dahuang has complex components, and its mechanism of action remains unclear [20–22]. Certain active ingredients such as emodin, chrysophanol, rhein, and physcion have been extracted from Dahuang and applied to treat hepatoma [23–26]. However, most studies on the antihepatoma mechanism of Dahuang are limited to the analysis of a single ingredient, and the pharmacodynamic mechanism and targets of Dahuang have not been described comprehensively and systematically.

**Figure 1:**
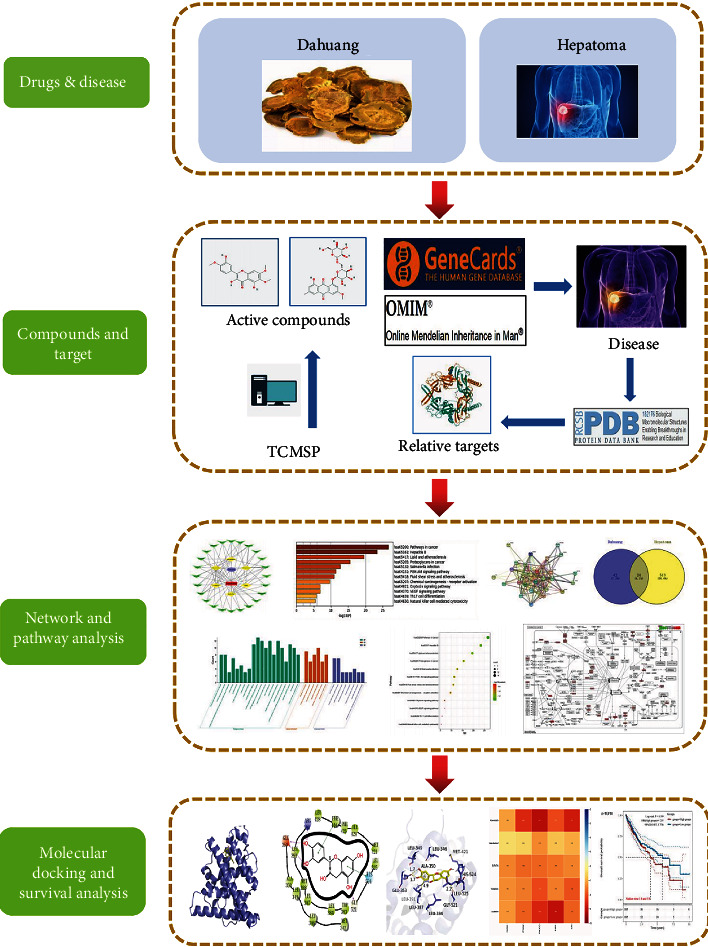
Technological roadmap for study.

Network pharmacology is based on multidisciplinary systems biology and multinomial pharmacology. The molecular mechanism of drug intervention in diseases is explored in a multidimensional form. It provides biological network construction and network visualization analysis for the complex interactions between drug ingredients and disease targets. This method is very suitable for the characteristics of multi-ingredient, multitarget, and multipathway action of TCM. It is widely used in studying ingredients and the mechanism of action of TCM [27]. Molecular docking is a computational method for predicting the binding interactions between small-molecule ligands and target proteins; it can confirm and test the results of network pharmacology [28]. Survival analysis examines and infers the survival time of hepatoma patients by expressing key target genes in hepatoma [29]. Additionally, the other in silico techniques, such as molecular dynamics simulation, molecular mechanics/generalized Born surface area model (MM/GBSA), and molecular force field/Poisson Boltzmann surface area model (MM/PBSA), can be used to screen inhibitors of key targets related to hepatoma. These technological methods have been widely used in the screening of key target protein inhibitors or agonists of related diseases in the natural product library of TCM [30–32]. They will play a role in promoting the modern research of TCM and the screening of new drug targets in the future. These methods are the focus of our later research. Currently, the aim of this study intends to use network pharmacology, molecular docking, and survival analysis for screening the effective active ingredients and explore potential targets for Dahuang against hepatoma and signal pathways involved. It will also investigate the critical target genes influencing the survival rate of hepatoma. The study will provide a theoretical basis and reference for new drug development, target mining, and animal experimentation in the future hepatoma's in-depth research. This research plan is shown in Figure 1.

## 2. Materials and Methods

### 2.1. Active Ingredients, Chemical Structures, and Potential Targets of Dahuang

The traditional Chinese medicine systems pharmacology (TCMSP) database and analysis platform (https://tcmsp-e.com/) were utilized to seek the active ingredients, chemical structure, and potential targets of Dahuang. According to the scheduling screening conditions, the oral bioavailability (OB) and druglikeness (DL) parameters were utilized to obtain the needed active ingredients, chemical structure, and potential targets. OB stands for the percentage of absorption of traditional Chinese medicine in the human circulatory system, and DL represents the similarity between the ingredients and known drugs [33]. “Dahuang” as the keyword was profoundly searched through the TCMSP database according to the screening condition values set as OB > 30% and DL > 0.18.

Although there are some exceptions, every active ingredient has its potential targets. Furthermore, the chemical structures of all active ingredients downloaded from the TCMSP were not in the figure format (TIFF, JPG, or PNG). Hence, the structures were redrawn using the ChemBioDraw Ultra software and then saved in the TIFF formats for better clarity.

### 2.2. The Potential Target Database of Hepatoma

The GeneCards database (https://www.genecards.org/) and Online Mendelian Inheritance in Man (OMIM database) (https://omim.org/) were used to seek the potential related targets of hepatoma. The keyword “Hepatoma” was used in these two databases. The relevant downloaded targets from both databases were merged and then deduplicated to get the potential targets. Further, the *R* software was employed to draw a Venn diagram for achieving the core targets between “Dahuang” and “Hepatoma.”

### 2.3. Construction of the Protein-Protein Interaction Network

The construction of the protein-protein interaction (PPI) network was completed on the STRING (https://string-db.org/) website. PPI can help us understand the functions and relationships of proteins [34]. On the STRING website, the core targets were entered into the “Multiple proteins” option, and “Homo sapiens” was selected in the organism query; the “default” criterion was established for other parameters. Finally, the PPI network was constructed.

### 2.4. Drug-Ingredient-Target-Disease Network Construction

The Cytoscape 3.7.2 software was used to construct a visualization network diagram regarding drug-ingredient-target-disease. The obtained drugs, ingredients, core targets, and diseases were/entered into the Cytoscape 3.7.2 software. A visualization network diagram was thus obtained from this software. Besides, the topological characteristics of “network nodes” were calculated through the Network Analyzer in the Cytoscape plug-in; these characteristics included different degrees of correlation, namely, degree, betweenness, and closeness. The degree value is a significant evaluation index, and the target showing a greater correlation with a disease can be identified [35]. Five core targets were acquired through the degree value of the software.

### 2.5. GO Functional Analysis and KEGG Enrichment Pathway Analysis

Gene Ontology (GO) functional enrichment analysis and Kyoto Encyclopedia of Genes and Genomes (KEGG) enrichment pathway analysis helped understand the function of the core targets and the critical pathways of Dahuang against hepatoma. The *R* software performed the GO function enrichment analysis and KEGG enrichment pathway analysis. The former results mainly included an amalgamation of biological processes, cellular components, and molecular function and were shown in a column chart. The larger the count value, the closer the function is to the core target. The KEGG enrichment pathway analysis results are shown in bubble diagram and histogram, with a *p* value; *p* < 0.05 indicated statistical significance.

### 2.6. Molecular Docking

The Maestro software was employed to achieve molecular docking between the five key targets in constructing the drug-ingredient-target-disease network and the main active ingredients of Dahuang [36]. The structures of the important target proteins were downloaded from the RCSB PDB protein structure database (https://www.rcsb.org/), and the chemical structures of the main active ingredients were obtained from the TCMSP platform. The screening criteria for PDB ID are based on the best score ranking of each structure in the PDB official website and the latest updated structure form. Furthermore, Maestro, PyMoL, and ChemBioDraw Ultra software were utilized to process the abovementioned protein receptors and ligands, and a molecular docking process was conducted to obtain the affinity among them. The results of molecular docking are presented in two-dimensional and three-dimensional graphics [37]. The present study used binding energy ≤ –5.0 kJ · mol–1 as the key criterion. The lower the binding energy, the better is the binding affinity between the target and the active ingredient [38]. In addition, we also used *R* software to draw a heat map based on the molecular docking results to analyze which active ingredients were more relevant to the target.

### 2.7. Survival Analysis

The impact of the main targets on the prognosis of hepatoma was analyzed through The Cancer Genome Atlas (TCGA) database (https://www.cancer.gov/about-nci/organization/ccg/research/structural-genomics/tcga). The core target gene expression was ranked from high to low, and high (50%) and low (50%) cut-off values were utilized as the expression thresholds to divide the two groups, namely, the high expression group and the low expression group. The low expression group had a better prognosis, and it relatively reflected that the specific gene might inhibit tumor development and progress; the opposite was considered a risk factor. Data analysis was performed using the *R* software. The log-rank test and Kaplan-Meier method analyzed 371 samples from the TCGA database to acquire survival maps; *p* < 0.05 was considered a statistically significant difference.

## 3. Results

### 3.1. The Collection of Active Ingredients, Chemical Structures, and Potential Targets

The screening conditions of the TCMSP database yielded 16 active Dahuang ingredients. Corresponding targets could not be identified for six of these active ingredients. Further, 66 related key targets were obtained for the remaining ten active ingredients. Since the chemical structures of the active ingredients were collected through the TCMSP database, their TIFF formats were redrawn by the ChemBioDraw Ultra software. The detailed information regarding the chemical structures is shown in Table 1.

### 3.2. The Related Targets of Hepatoma

**Figure 2:**
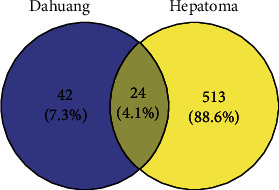
Venn diagram.

The related targets of hepatoma were acquired through GeneCards and OMIM database. For hepatoma, 528 related targets were received from the GeneCards database, and only the targets with a correlation score ≥ 1.0 were included. Additionally, 19 related hepatoma-related targets were obtained from the OMIM database. After merging the two datasets, deduplication was performed, and 537 hepatoma-related targets were obtained. Further, the 66 targets of Dahuang were combined with the 537 targets of hepatoma. The Venn diagram was drawn, and the intersection was identified (Figure 2). Finally, 24 common action targets of Dahuang and hepatoma were obtained. These targets are nitric oxide synthase 2 (NOS2), androgen receptor (AR), prostglandin-endoperoxide synthase 2 (PTGS2), coagulation factor II, thrombin (F2), kinase insert domain receptor (KDR), phosphatidylinositol-4,5-bisphosphate 3-kinase catalytic subunit gamma (PIK3CG), JUN protooncogene, AP-1 transcription factor subunit (JUN), estrogen receptor 1 (ESR1), BCL2 apoptosis regulator (BCL2), peroxisome proliferator activated receptor gamma (PPARG), Fas cell surface death receptor (FAS), caspase 9 (CASP9), caspase 3 (CASP3), caspase 8 (CASP8), protein kinase C alpha (PRKCA), transforming growth factor beta 1 (TGFB1), paraoxonase 1 (PON1), interleukin 1 beta (IL1B), cyclin-dependent kinase inhibitor 1A (CDKN1A), tumor necrosis factor (TNF), tumor protein P53 (TP53), proliferating cell nuclear antigen (PCNA), MYC protooncogene, BHLH transcription factor (MYC), and heat shock protein 90 alpha family class A member 1 (HSP90AA1).

### 3.3. The Construction of PPI

**Figure 3:**
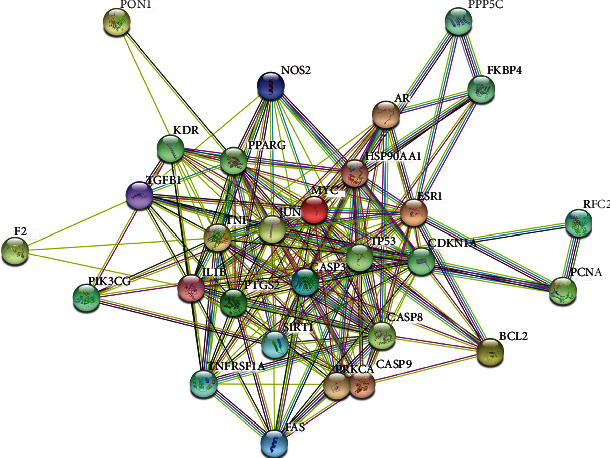
PPI network map.

The interaction datasets were acquired by incorporating 24 core targets into the STRING platform and selecting “Homo sapiens” to generate the PPI network map. Subsequently, the associated protein interaction relationships were also obtained (Figure 3). In the map, 24 inherent nodes and 158 edges existed. Each node represented a different protein; the edges represented their innate relationships, while the colors from yellow to red represented small to large values. Additionally, a connection between proteins was indicated by a line between them. The greater the number lines between the proteins, the closer is the relationship between them.

### 3.4. The Construction of the Drug-Ingredient-Target-Disease Network

**Figure 4:**
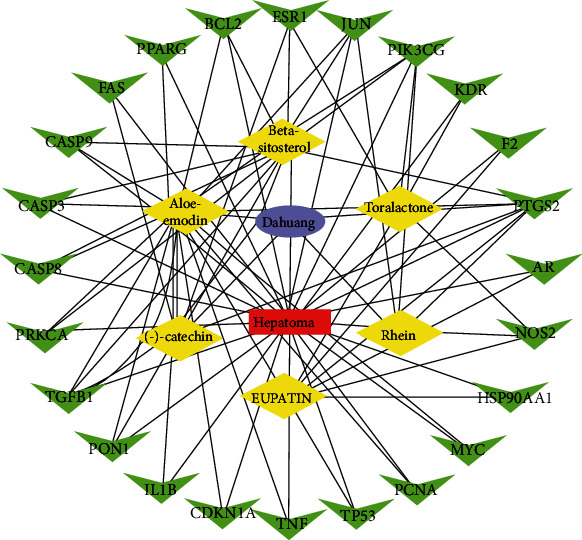
Drug-ingredient-target-disease network diagram.

The Dahuang-ingredient-target-hepatoma network diagram was constructed by Cytoscape 3.7.2 software (Figure 4). The active ingredients and maximum key targets had numerous interactions as deduced through the computational inference. This implied that Dahuang worked against hepatoma through a precise analysis of multi-ingredient, multitarget, and multipathway synergistic interactions. More importantly, Dahuang displayed a tremendous potential influence on hepatoma therapy. Utilizing the Network Analyzer of Cytoscape 3.7.2 software, a degree analysis was performed wherein five main Dahuang ingredients, and five key targets were screened. The five main active ingredients were aloe-emodin, beta-sitosterol, eupatrid, toralactone, and (-)-catechin, while the five key targets were transforming growth factor beta 1 (TGFB1), prostglandin-endoperoxide synthase 2 (PTGS2), phosphatidylinositol-4,5-bisphosphate 3-kinase catalytic subunit gamma (PIK3CG), estrogen receptor 1 (ESR1), and protooncogene, AP-1 transcription factor subunit (JUN).

### 3.5. GO and KEGG Enrichment Analysis

**Figure 5:**
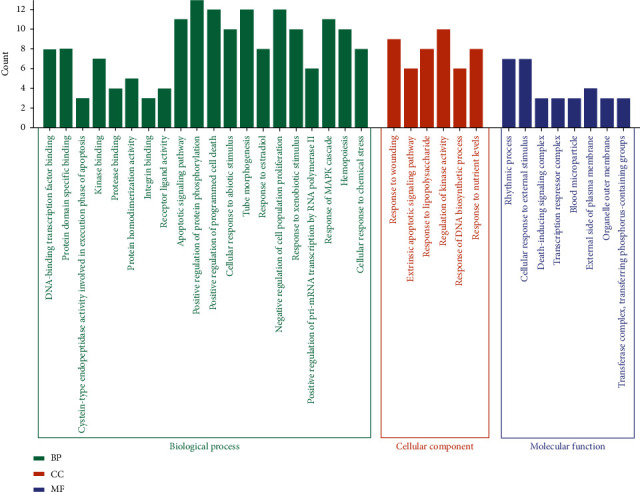
The results of GO functional enrichment analysis.

The *R* software explored the possible mechanisms of the 24 candidate targets for the treatment of hepatoma. A total of 34 related biological processes, molecular functions, and cellular components were obtained with a significance value of *p* < 0.05 (Figure 5). The coaction targets were mainly enriched in the positive regulation of protein phosphorylation, positive regulation of programmed cell death, tube morphogenesis, negative regulation of cell population proliferation, regulation of kinase activity, rhythmic process, cellular response to external stimulus, etc.

**Figure 6:**
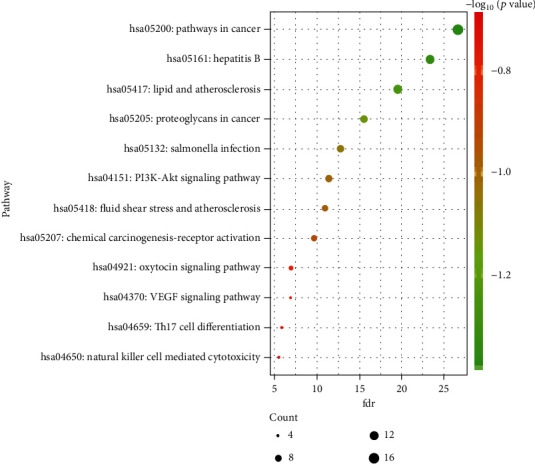
Bubble diagrams of the KEGG pathway enrichment analysis.

**Figure 7:**
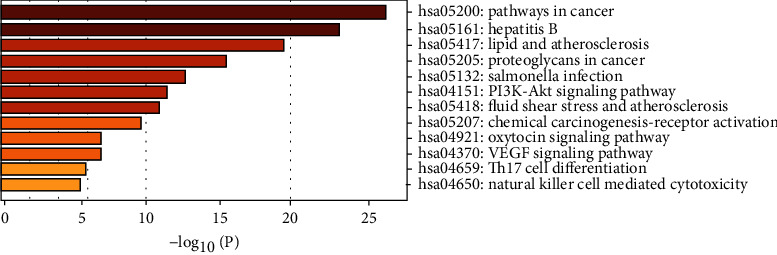
The results of KEGG enrichment analysis.

**Figure 8:**
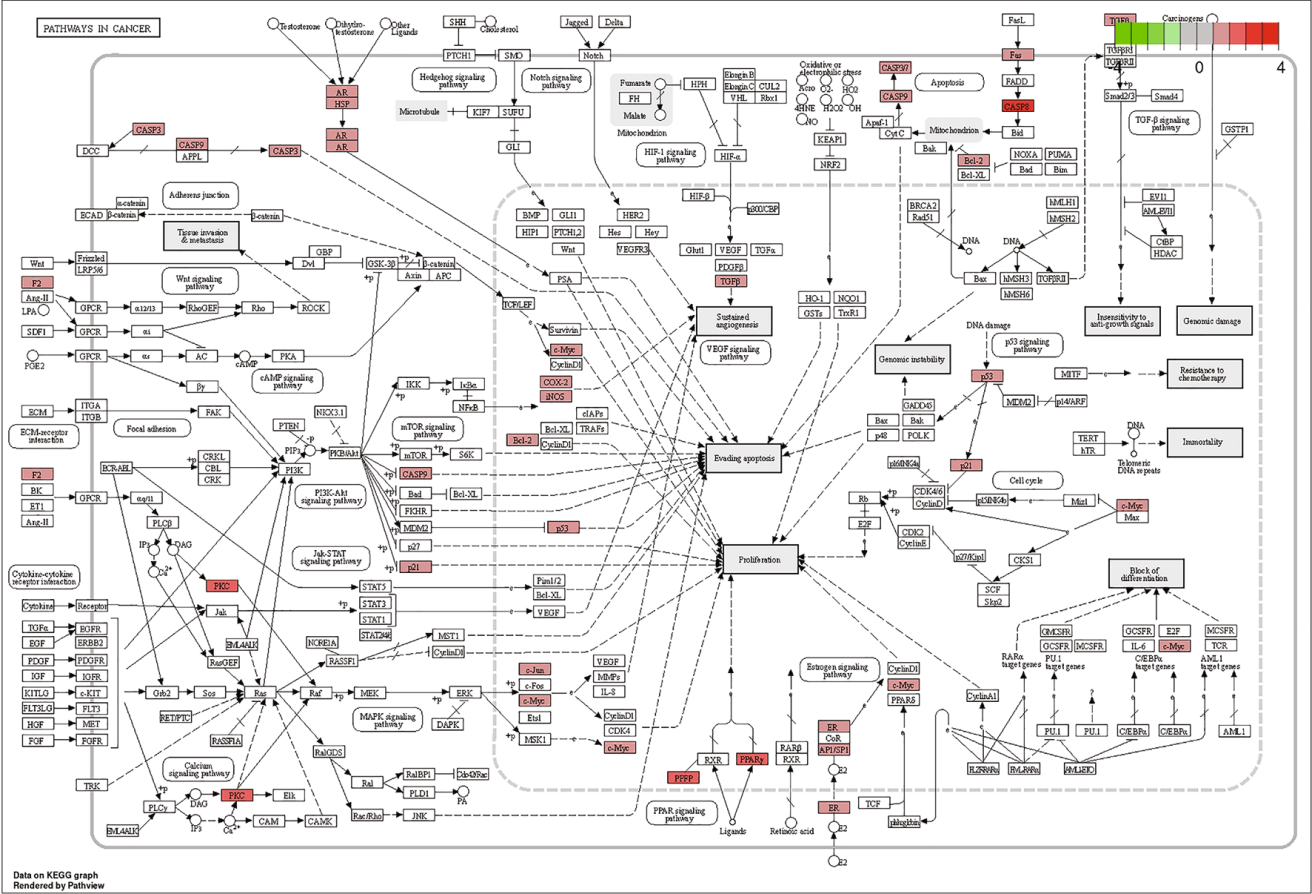
The anti hepatoma pathway of Dahuang.

The KEGG enrichment analysis was performed to explore the possible mechanisms of the 24 candidate targets for hepatoma treatment; a total of 12 main items with *p* < 0.05 were obtained (bubble chart and histogram in Figures 6 and 7). The coaction targets were mainly enriched in several diseases and some pathways: pathways in cancer, hepatitis B, lipid and atherosclerosis, proteoglycans in cancer, Salmonella infection, PI3K-Akt signaling pathway, fluid shear stress, atherosclerosis, chemical carcinogenesis-receptor activation, oxytocin signaling pathway, VEGF signaling pathway, Th17 cell differentiation, and natural killer cell-mediated cytotoxicity. Thus, the critical related targets in Dahuang mechanism and pathways in cancer were identified (Figure 8).

### 3.6. Molecular Docking

In this experiment, five compounds (aloe-emodin, beta-sitosterol, eupatin, toralactone, and (-)-catechin) were docked with ESR1, JUN, PIK3CG, PTGS2, and TGFB1 target proteins. Aloe-emodin, beta-sitosterol, eupatin, toralactone, and (-)-catechin exhibited good binding and high matching degree with the five target proteins. Beta-sitosterol performed the worst, mainly because it is fat-soluble and exhibits strong hydrophobicity. Moreover, the molecule is large and not conducive to matching the protein pocket. The complex formed between the docking compound and the protein was visualized with PyMoL 2.1 software (the combination with the most negative docking score was selected for each target), and the binding mode of the compound and the protein was obtained. According to the binding mode, the amino acid residues of the compound and the protein pocket combination were clearly seen.

**Figure 9:**
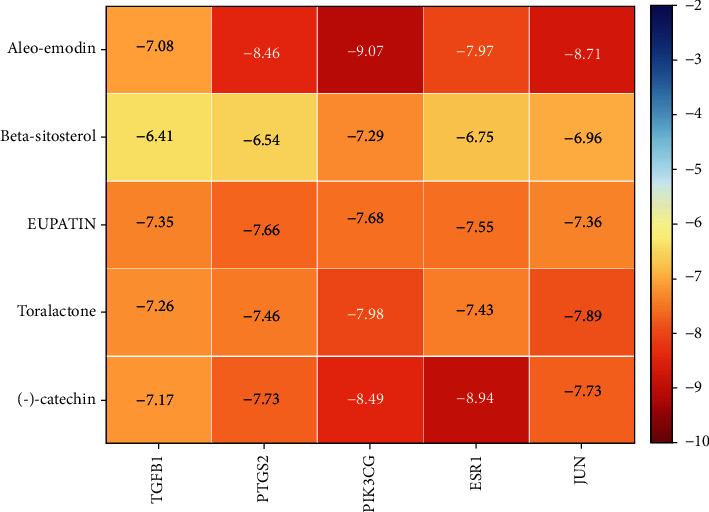
The heat map of molecular docking results.

For example, the amino acid residues of JUN interacting with the active sites of aloe-emodin included LEU-855, VAL-863, ALA-880, MET-929, GLU-930, VAL-911, GLY-993, LEU-983, SER-936, LEU-932, ASP-939, and TYR-931. Aloe-emodin is a strongly hydrophobic anthraquinone compound. It forms a strong hydrophobic interaction with active site amino acids (LEU-855, VAL-863, ALA-880, MET-929, VAL-911, LEU-983, LEU-932, TYR-931) and plays an essential role in stabilizing the small molecules in the protein cavity. Additionally, the compound forms multiple hydrogen bonds with ASP-939, LEU-932, and GLU-930 amino acids; this bonding contributes considerably to anchor small molecules in the protein cavity. Several other compounds also exhibit hydrogen bonding and hydrophobic interactions with ESR1, PIK3CG, PTGS2, and TGFB1 targets; such interactions effectively promote the formation of stable complexes between small molecules and proteins. In conclusion, these compounds (aloe-emodin, beta-sitosterol, eupatin, toralactone, and (-)-catechin) and the target proteins (ESR1, JUN, PIK3CG, PTGS2, and TGFB1) performed well in the docking scoring and binding mode. These compounds formed stable complexes with proteins and correlated strongly with the five targets. The detailed results of molecular docking is seen in Table 2. The heat map based on the results of molecular docking is seen in Figure 9, which indicated that the redder the color of the square, the closer the binding between the active ingredient and the target.

### 3.7. Survival Analysis

**Figure 10:**
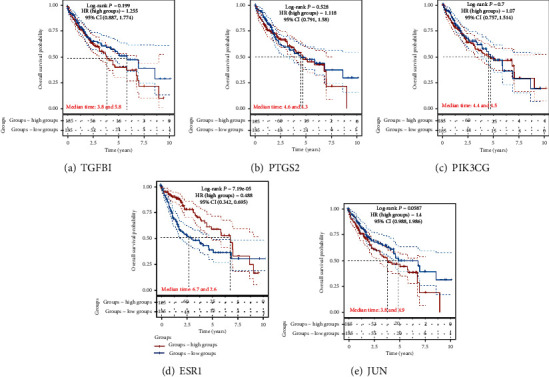
Survival analysis for 5 main genes by TCGA.

All cases of hepatoma were categorized into high and low expression groups based on the expression levels of the five core genes. The correlation between the expression of the individual core genes and the prognosis of the hepatoma patients was analyzed by exploring the TCGA database. The genes with lower expression were associated with a poor prognosis. ESR1 was associated with overall survival in all hepatoma patients (*p* = 7.19*e*^–5^), indicating that it may inhibit the development and progress of hepatoma. The overall survival analysis of the remaining four core genes with high and low expression displayed no statistical significance (*p* < 0.05) (Figure 10).

## 4. Discussion

In recent years, with advancements in traditional Chinese medicine and the continuous improvement of the technical field, traditional Chinese medicine has reached new heights. Traditional Chinese medicine exhibits various unique properties like varied active substances, multiple key targets, and low toxicity [39]. More importantly, it has superior antitumor properties and can be used to treat different tumors, such as esophageal cancer, liver cancer, pancreatic cancer, and breast cancer [40–43]. The present study identified the key active ingredients and possible detailed molecular mechanisms of Dahuang against hepatoma through network pharmacology, molecular docking, and survival analysis. The use of Dahuang is expected to improve the therapeutic effect further and prolong survival in hepatoma patients. In this study, the active ingredients and related targets of Dahuang were screened from the TCMSP database. The potential targets of hepatomas were further analyzed by the GeneCards and OMIM databases. Additionally, intersections of Dahuang-related targets and hepatoma-related targets revealed common core targets. Subsequently, PPI network and drug-ingredient-target-disease network were constructed through core targets, and then GO function and KEGG pathway enrichment analysis were performed by using *R* software. The top five active Dahuang ingredients involved in degree analysis were aloe-emodin, beta-sitosterol, eupatin, toralactone, and (-)-catechin, while the top five core targets were ESR1, JUN, PIK3CG, PTGS2, and TGFB1. The combination of aloe-emodin and PIK3CG exhibited the best binding affinity (-9.07 kJ·mol^–1^). Besides, ESR1 was found to inhibit the development and progress of hepatoma.

Dahuang contains five main active ingredients: aloe-emodin, beta-sitosterol, eupatin, toralactone, and (-)-catechin. Aloe-emodin (1,8-dihydroxy-3-hydroxymethyl-9,10-anthrenedione) is an anthraquinone found in traditional medicinal plants such as Dahuang. The early studies of the 21^st^ century proved the antihepatoma effect of aloe-emodin [44]. Aloe-emodin induced p53 expression and p21 expression in Hep G2 cells, related to G1 cell cycle arrest. In p53-deficient Hep 3B cells, aloe-emodin inhibited cell proliferation in a p21-dependent manner. In addition, some scholars demonstrated that aloe-emodin inhibited the proliferation of the human hepatoma cell line HuH-7 and played an antihepatoma role [45]. These findings suggested that aloe-emodin might help prevent hepatoma. Vo et al. found that beta-sitosterol exhibited cytotoxicity against HepG2 and HuH-7 cells but not against normal human primary fibroblasts [46]. Beta-sitosterol inhibited the proliferation of the HepG2 and HuH-7 cells in a dose-dependent manner: its maximum half-maximal inhibitory concentration (IC_50_) values were 6.85 ± 0.61 *μ*g/mL and 8.71 ± 0.21 *μ*g/mL, respectively (*p* < 0.01). Moreover, beta-sitosterol exhibited cytotoxic activity by inducing apoptosis and activating caspase-3 and caspase-9 in these cells. These findings suggested that beta-sitosterol may be developed into a promising therapeutic agent for treating hepatoma. Eupatilin, a lipophilic flavonoid, is an agonist of PPAR*α* and has antiapoptotic, antioxidant, and anti-inflammatory effects [47]. Presently, there is no clarity regarding its antitumor effect. Toralactone is a natural product isolated from *Cassia obtusifolia*. It mediates hepatoprotection via an Nrf2-dependent antioxidative mechanism. However, its anticancer action is not known [48]. Catechin has good antitumor effects against gastric cancer, cervical cancer, pancreatic cancer, and lung cancer. However, two of its isomers have different functions [49–52]. The four isomers of catechin are (+)-catechin, (-)-catechin, (+)-epicatechin, and (-)-epicatechin. Among them, (+)-catechin has an anticancer effect, while the anticancer effect of (-)-catechin has not been identified yet. However, it inhibits COX-1 and may also have a potential antitumor impact that needs to be further confirmed in the future [53, 54].

The drug-ingredient-target-disease network and degree ranking identified five core genes that included ESR1, JUN, PIK3CG, PTGS2, and TGFB1. These genes play an essential role in tumor development, particularly in the process of proliferation, migration, and apoptosis. The network analysis and survival analysis results identified ESR1 as an essential protective factor in hepatoma treatment. There was no significant difference in the survival analysis of other proteins in hepatoma patients. ESR1 has also been reported as a candidate tumor suppressor gene for hepatoma [55]. The reduced ESR1 expression is associated with a high liver injury score, tumor size, and pathological invasion of the intrahepatic portal vein [56]. HCC patients with low ESR1 expression also have a shorter survival period [57]. Mir-9–5P, an oncogene, promotes the proliferation, migration, and invasion of HCC cells by inhibiting the ESR1 expression [58]. The present results are also similar to the results of Meng et al. wherein a higher expression of ESR1 inhibited the occurrence and development of hepatoma.

The GO functional enrichment analysis inferred some significant biological processes, cellular compositions, and intermolecular functions in the core targets (Figure 5). The KEGG enrichment analysis revealed the pathways involved in cancer, such as the PPAR signaling pathway, MAPK signaling pathway, calcium signaling pathway, cAMP signaling pathway, cytokine-cytokine receptor interaction, HIF-1 signaling pathway, p53 signaling pathway, and mTOR signaling pathway (Figure 8). Therefore, these pathways are associated with hepatoma. The molecular docking exercise revealed the greatest affinity between aloe-emodin and PIK3CG. These results indicated that PIK3CG might be the critical target for the action of Dahuang against hepatoma. However, a previous study had demonstrated the importance of PIK3CG in the progression and metastasis of prostate cancer rather than in hepatoma. PIK3CG may represent a new therapeutic target in metastatic castration-resistant prostate cancer [59]. Hence, its role in hepatoma remains unclear. In the current studies, the exploration and further value of PIK3CG in hepatoma may be lower than ESR1. Furthermore, aloe-emodin and beta-sitosterol may also be effective ingredients for the potential treatment of hepatoma.

The present study was based on public databases, which had restricted information, and needed continuous improvement. Besides, the study also ignored the clinical use of Dahuang, such as drug dose, medication frequency, and administration method. However, this study is also worthy of further exploration and verification to extract the intricate molecular mechanisms governing the therapeutic targets for hepatoma therapy.

## 5. Conclusion

Based on the network pharmacology, molecular docking, and survival analysis, our study results predicted the critical active ingredients and core targets of Dahuang. Further, it explored the potential targets of hepatoma to design effective drug therapies for the future. The GO and KEGG enrichment analysis revealed that the possible working mechanism of Dahuang against hepatoma mainly focused on positive regulation of protein phosphorylation, regulation of kinase activity, rhythmic process, and cellular response to external stimulus. Moreover, a few important observations were drawn. Pathways in cancer and its related pathways are the main pathways of Dahuang against hepatoma. ESR1 is the potential key gene that is beneficial for the survival of hepatoma patients. Also, aloe-emodin and beta-sitosterol are the two main active ingredients crucial for hepatoma treatment. Nevertheless, this study needs further experimental verification to determine the exact situation of Dahuang and its active ingredients in the treatment of hepatoma. Later, the current team might conduct a pharmacological study on critical signaling pathways and targets of Dahuang against hepatoma to verify it.

## Figures and Tables

**Table 1:** Information of active ingredients and related targets from Dahuang.

mol ID	mol name	mol structure	Related targets	OB (%)	DL
MOL002235	Eupatin	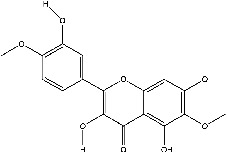	NOS2, AR, F10, PTGS2, F8, TOP2, ESR2, DPP4, HSP90AB1, HSP90AA1, PRSS1, NCOA2, CALM1, F2, SCN5A, KDR, PPARD	50.80	0.41
MOL002259	Physciondiglucoside	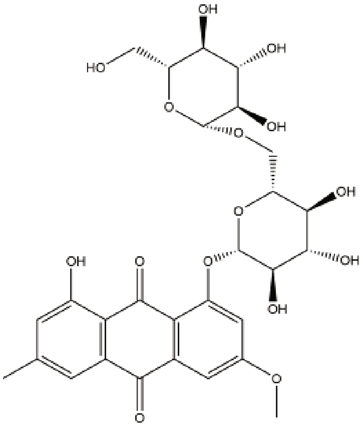	TOP2	41.65	0.63
MOL002268	Rhein	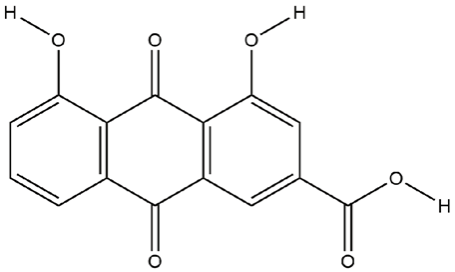	PTGS1, PTGS2, HSP90AB1, HSP90AA1, PIK3CG, NCOA2, AKR1B1, JUN	47.07	0.28
MOL002280	Torachrysone-8-O-beta-D-(6′-oxayl)-glucoside	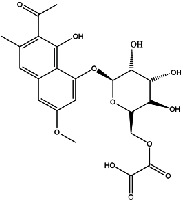	TOP2	43.02	0.74
MOL002281	Toralactone	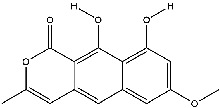	NOS2, PTGS1, ESR1, PTGS2, ESR2, HSP90AB1, HSP90AA1, PIK3CG, CHEK1	46.46	0.24
MOL002288	Emodin-1-O-beta-D-glucopyranoside	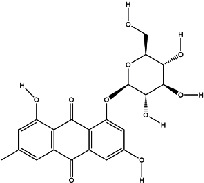	TOP2	44.81	0.80
MOL002297	Daucosterol_qt	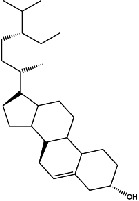	PGR, NCOA2	35.89	0.70
MOL000358	Beta-sitosterol	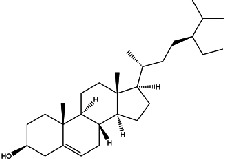	PGR, NCOA2, PTGS1, PTGS2, HSP90AB1, HSP90AA1, PIK3CG, KCNH2, DRD1, CHRM3, CHRM1, SCN5A, GABRA2, CHRM4, PDE3A, HTR2A, GABRA5, ADRA1A, GABRA3, CHRM2, ADRA1B, ADRB2, CHRNA2, SLC6A4, OPRM1, GABRA1, CHRNA7, BCL2, CASP9, JUN, CASP3, CASP8, PRKCA, TGFB1, PON1, MAP2	36.91	0.75
MOL000471	Aloe-emodin	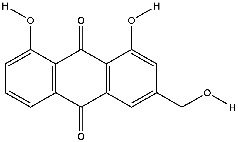	PTGS1, PTGS2, HSP90AB1, HSP90AA1, PIK3CG, NCOA2, PKIA, AKR1B1, IGHG1, CDKN1A, EIF6, TNF, CASP3, TP53, FAS, PRKCA, PRKCE, CRK2, PCNA, MYC, IL1B, PRKCD	83.38	0.24
MOL000096	(-)-Catechin	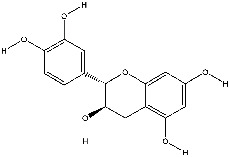	PTGS1, ESR1, PTGS2, HSP90AB1, HSP90AA1, ampC, NCOA2, CALM1, FAS, PPARG	49.68	0.24

**Table 2:** Molecular docking results of 5 main active ingredients and 5 core targets.

Target	PDB ID	Target structure	Active ingredients	Affinity (kJ·mol^−1^)	Best-docked complex (3D) and (2D)	Interacting residues	Type of interactions
TGFB1	5VQP	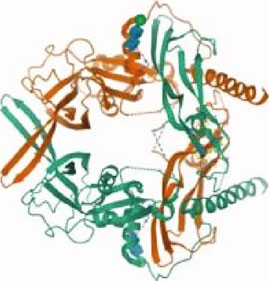	Aloe-emodin	-7.08	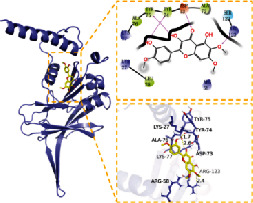	ALA 76, TYR 75, TYR 74, ASP 73, ALA 72, ARG 123, ARG 58, LEU 28, LYS 27, LYS 77	Hydrophobic interaction, hydrogen bond interactions
			Beta-sitosterol	-6.41			
			Eupatin	-7.35			
			Toralactone	-7.26			
			(-)-Catechin	-7.17			
PTGS2	5IKR	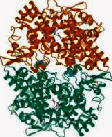	Aloe-emodin	-8.46	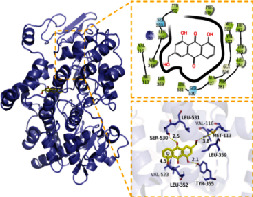	PHE 518, MET 522, VAL 523, GLY 526, ALA 527, LEU 531, LEU 359, MET 113, VAL 116, ARG 120, VAL 349, LEU 352, YTR 355, TRP 387, TYR 385, LEU 384, PHE 381	Hydrophobic interaction, hydrogen bond interactions
			Beta-sitosterol	-6.54			
			Eupatin	-7.66			
			Toralactone	-7.46			
			(-)-Catechin	-7.73			
PIK3CG	6AUD	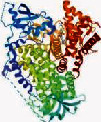	Aloe-emodin	-9.07	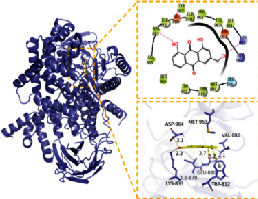	ALA 885, VAL 882, ILE 881, GLU 880, TYR 867, ILE 879, PHE 961, ILE 962, ASP 964, HIP 967, ILE 831, LYS 883, MET 804, PRO 810, TRP 812, MET 963	Hydrophobic interaction, hydrogen bond interactions
			Beta-sitosterol	-7.29			
			Eupatin	-7.68			
			Toralactone	-7.98			
			(-)-Catechin	-8.49			
ESR1	7KCD	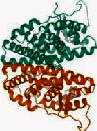	Aloe-emodin	-7.97	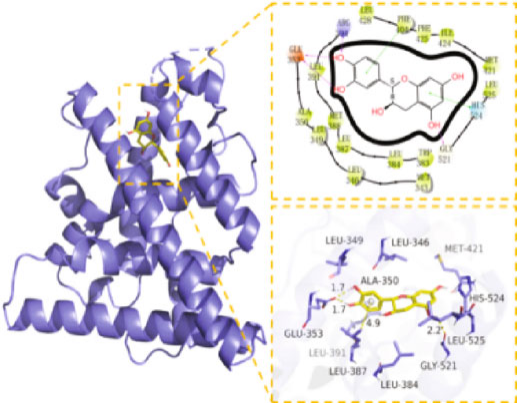	MET 929, GLU 930, TYR 931, LEU 932, GLY 935, ASP 939, GLY 856, LEU 855, VAL 863, LEU 983, ASP 994, GLY 993, VAL 911, ALA 880	Hydrophobic interaction, hydrogen bond interactions
			Beta-sitosterol	-6.75			
			Eupatin	-7.55			
			Toralactone	-7.43			
			(-)-Catechin	-8.94			
JUN	5AEP	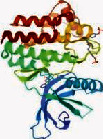	Aloe-emodin	-8.71	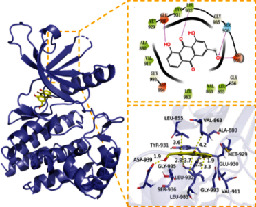	LEU 428, PHE 404, PHE 425, ILE 424, MET 421, LEU 525, GLY 521, TRP 383, LEU 384, MET 388, LEU 391, ARG 394, MET 343, LEU 346, LEU 349, ALA 350, GLU 353	Hydrophobic interaction, hydrogen bond interactions
			Beta-sitosterol	-6.96			
			Eupatin	-7.36			
			Toralactone	-7.89			
			(-)-Catechin	-7.73			

## Data Availability

The data that support the findings of this study are available on request from the corresponding author.

## Abbreviations

DL: Druglikeness
GO: Gene Ontology
HCC: Hepatocellular carcinoma
KEGG: Kyoto Encyclopedia of Genes and Genomes
OB: Oral bioavailability
OMIM: Online Mendelian Inheritance in Man
PPI: Protein-protein interaction
TCM: Traditional Chinese medicine
TCMSP: Traditional Chinese Medicine Systems Pharmacology
TCGA: The Cancer Genome Atlas.
